# Individual Differences in Impulsivity Predict Head Motion during Magnetic Resonance Imaging

**DOI:** 10.1371/journal.pone.0104989

**Published:** 2014-08-22

**Authors:** Xiang-zhen Kong, Zonglei Zhen, Xueting Li, Huan-hua Lu, Ruosi Wang, Ling Liu, Yong He, Yufeng Zang, Jia Liu

**Affiliations:** 1 State Key Laboratory of Cognitive Neuroscience and Learning & IDG/McGovern Institute for Brain Research, Beijing Normal University, Beijing, China; 2 School of Psychology, Beijing Normal University, Beijing, China; 3 Center for Collaboration and Innovation in Brain and Learning Sciences, Beijing Normal University, Beijing, China; 4 Center for Cognition and Brain Disorders, Hangzhou Normal University, Hangzhou, China; Duke-NUS Graduate Medical School, Singapore

## Abstract

Magnetic resonance imaging (MRI) provides valuable data for understanding the human mind and brain disorders, but in-scanner head motion introduces systematic and spurious biases. For example, differences in MRI measures (e.g., network strength, white matter integrity) between patient and control groups may be due to the differences in their head motion. To determine whether head motion is an important variable in itself, or just simply a confounding variable, we explored individual differences in psychological traits that may predispose some people to move more than others during an MRI scan. In the first two studies, we demonstrated in both children (N  =  245) and adults (N  =  581) that head motion, estimated from resting-state functional MRI and diffusion tensor imaging, was reliably correlated with impulsivity scores. Further, the difference in head motion between children with attention deficit hyperactivity disorder (ADHD) and typically developing children was largely due to differences in impulsivity. Finally, in the third study, we confirmed the observation that the regression approach, which aims to deal with motion issues by regressing out motion in the group analysis, would underestimate the effect of interest. Taken together, the present findings provide empirical evidence that links in-scanner head motion to psychological traits.

## Introduction

Head motion during an MRI scan is undesirable, which not only displaces the brain in space, but also interferes with the MR signals [1], [2], [3], [4], [5]. Recent studies have further discovered that head motion introduces spurious biases [6], [7], [8], [9], [10] and poses difficulty in identifying signatures of neurological disorders, including attention deficit hyperactivity disorder (ADHD) and autism [8], [11]. For example, in-scanner head motion decreases long-range functional connectivity [5],[7]. Therefore, it is possible that the reduced functional connectivity between the medial prefrontal cortex (MPFC) and posterior cingulate cortex (PCC) observed in ADHD [12], [13], [14] may be largely due to more intensive head motion of patients with ADHD during scanning. Because head motion significantly confounds both MRI data acquisition and interpretation, it is necessary to characterize the nature of the motion: what makes people move their head during scanning, and why some people move more than others do?

One possible reason of head motion is that some individuals are unable to control their behaviors during scanning. Indeed, MRI data from patients with ADHD and autism contained more volumes with excessive head motion (e.g., > 2 mm) [15], [16], implying that they move more intensively than healthy controls. Greater head motion is also found in neurological disorders such as multiple sclerosis [17]. Although some studies did not observe greater head motion in mental disorders such as schizophrenia (e.g., [18]), the failure may be due to low disease severity and small sample size [17]. In addition, the magnitude of head motion is reliable across multiple scans within an individual and varies significantly across individuals [8], [9], suggesting that head motion might reflect a trait-like property of human participants. Importantly, a recent study shows that head motion during brain imaging reflects a neurobiological trait rather than simply a technical artifact, suggesting that head motion may be an indicator of a specific cognitive control capacity in the individual brain [19]. Taken together, we hypothesize that the psychological trait of impulsivity is a potential factor contributing to head motion during MRI scan, with impulsive individuals moving their head more intensively in an MRI scanner.

To test this hypothesis, we examined the relationship between head motion, measured during scanning, with the psychological trait of impulsivity measured outside the scanner. In Study 1, we examined whether individual differences in impulsivity assessed by Barratt impulsiveness scale (BIS) [20] significantly accounted for the variance in head motion. Thus, in Study 2, we investigated whether group differences in head motion between children with ADHD and typically developing children (TDC) reflected differences in impulsivity. All these results converged to demonstrate that head motion contains information regarding an individual's impulsivity and, thus, it provides a novel perspective in understanding motion effects in MRI data analyses and interpretation. Further, given that head motion was correlated with impulsivity, regressing out head motion in across-subject analyses as suggested by some studies [8], [21] would result in the underestimation of the effect of interest. The final study confirmed this hypothesis by showing that simply regressing out head motion significantly reduced the association between the fMRI metric (i.e., amplitude of low frequency fluctuation, ALFF) and impulsivity.

## Materials and Methods

### 2.1 Participants

Five hundred and eighty-one college students (mean age: 20.5 years; SD: 0.95; 327 females) from Beijing Normal University (BNU), Beijing, China, participated in Study 1. The dataset is part of the Brain Activity Atlas Project (BAAP, http://www.brainactivityatlas.org/). Participants reported no past or current psychiatric illness or history of neurological disorders. Both behavioral and MRI protocols were approved by the Institutional Review Board of Beijing Normal University. Written informed consent was obtained from all participants prior to the experiment. Fifteen outlier participants (2.6% of all participants) were excluded from further analyses; outliers were defined as being 2 SD below or above the group mean for head motion (see *Assessment of In-Scanner Head Motion*).

Participants for Study 2 and 3 included both ADHD and TDC from the ADHD-200 Consortium (http://fcon_1000.projects.nitrc.org/indi/adhd200/). Because the data were from multiple imaging centers, we only analyzed data collected from our imaging center to minimize variability across centers. This sub-dataset contained 245 children, 102 of whom were diagnosed with ADHD (mean age: 12.08 years, SD: 2.04; 12 females); the remaining 143 participants were TDC (mean age: 11.43 years, SD: 1.86; 59 females). Details on the inclusion criteria are available at the website of ADHD-200. Two children with ADHD (2% of the ADHD population) and 3 TDC (2% of the TDC population) showed excessive head motion (i.e., greater than 2 SD above the group mean of head motion for each group); therefore, were excluded from further analyses. Behavioral measures of hyperactivity/impulsivity were not available for 7 children with ADHD and 16 TDC; therefore, they were not included in further analyses either.

### 2.2 Assessment of Impulsivity

#### 2.2.1 Barratt impulsiveness scale (BIS)

In Study 1, impulsivity was assessed using the BIS [20], which was a 4-point Likert-type scale containing 30 items. In the BIS, higher values indicate greater levels of impulsivity. In addition to the total score as a comprehensive measure of impulsivity, BIS contains six components of impulsivity: attention (focusing on task at hand), cognitive instability (thought insertions and racing thoughts), motor (acting on the spur of the moment), perseverance (a consistent life style), self-control (planning and thinking carefully), and cognitive complexity (enjoying challenging mental tasks).

#### 2.2.2 The ADHD Rating Scale (ADHD-RS) IV

In Study 2, the ADHD-RS IV was used to assess ADHD symptoms. This consists of a 4-point Likert-type scale containing 18 items, with each item corresponding to one of the 18 DSM-IV diagnostic criteria. Higher scores indicated greater ADHD-related behavior. ADHD-RS IV has 2 subscales: inattentive (difficulties in focusing on one thing) and hyperactivity/impulsivity (difficulties in behavioral inhibition). We focused on the hyperactivity/impulsivity subscale on the basis of our findings in Studies 1.

### 2.3 Assessment of In-Scanner Head Motion

#### 2.3.1 Assessment of In-Scanner Head Motion from Diffusion Tensor Images

Diffusion tensor (DT) images were collected on a 3T scanner (Siemens Trio, with a Tim system) at the BNU Imaging Center for Brain Research, Beijing, China. Diffusion-weighted images were collected using a single-shot spin-echo echo-planar imaging (SE-EPI) sequence: repetition time (TR)  =  7.2 s, echo time (TE)  =  104 ms, flip angle  =  90°, field of view (FOV)  =  230 mm, matrix size  =  128 × 128 mm, resolution  =  1.8 × 1.8 × 2.5 mm^3^, 49 slices, slice thickness  =  2.5 mm. The diffusion weighting gradients were applied along 64 non-collinear directions using a b-value of 1000 s/mm^2^, together with an acquisition without diffusion weighting (b  =  0 s/mm^2^). The total scan time was 7 min 48 s. Foam padding was used to restrict motion within the scanner.

DTI images were processed with FMRIB's Software Library (FSL, http://www.fmrib.ox.ac.uk/fsl). Volumes were realigned to the first volume with a rigid body transform correction with FMRIB's Linear Image Registration Tool (FLIRT), and rigid transformation matrices were obtained for each volume. To estimate in-scanner head motion, the root-mean-square (RMS) deviation, which summarizes 6 translations and rotations across 3 axes, was calculated from 2 transformations of 2 consecutive volumes [3]. That is, in-scanner head motion was measured as the summary measure of both translations and rotations of each brain volume relative to the preceding one as previous studies [8], [9]. Finally, head motion was calculated by averaging the RMS deviations for all volumes.

#### 2.3.2 Assessment of In-Scanner Head Motion from Resting-State functional MR Images

The resting-state fMRI data were from the ADHD-200 Consortium. Brain images were collected using a T2*-weighted gradient-echo EPI (GRE-EPI) sequence: TR  =  2 s, TE  =  30 ms, flip angle  =  90°, FOV  =  220 mm, matrix size  =  64 × 64, 30 axial slices, slice thickness  =  4.5 mm. Participants were instructed to keep their eyes closed and to relax during the scan. The total scan time lasted 8 min. Foam padding was used to restrict head motion within the scanner.

In-scanner head motion was calculated from the resting-state fMRI images using the same procedures described in Study 1.

### 2.4 Relationship between In-Scanner Head Motion and Impulsivity

#### 2.4.1 Correlation Analysis

Pearson correlation analyses were used to examine the potential link between head motion and impulsivity scores in both adult and child datasets. In Study 1, the correlation between head motion and impulsivity total score was calculated. Given the multi-dimensional property of impulsivity [22], [23], [24], we further investigated which components of impulsivity [20] mainly contributed to the significant association. To account for multiple testing with these multiple components, we used the Bonferroni correction and considered significant only components for which p < 0.05/6. In addition, to ensure the findings robust to data non-normality and to avoid the influence of outliers, Spearman's rank-correlation coefficient was also calculated for each correlation analysis.

#### 2.4.2 Mediation Analysis with Dataset from Children with or without ADHD

In this study, we used the mediation analysis [25], [26] in order to test the mediation effect of impulsivity in the ADHD-TDC group difference in head motion. In other words, we aimed to test whether the difference between groups in the trait of impulsivity accounted for the difference in head motion between children with ADHD and children in the TDC group. In the mediation model, “a” represents the relation of an independent variable (i.e., X, or ADHD versus TDC) to a mediating variable (i.e., M, or impulsivity), “b” represents the relation of M to a dependent variable (i.e., Y, or head motion), “total” represents the relation of X to Y, and “direct” represents the relation of X to Y after being adjusted for M. The mediated effect may be calculated in two ways, as either “a*b” or “total – direct,” both of which correspond to the reduction in the effect of X on Y when M was adjusted (for a review, see [26]). In this study, we conducted the mediation analysis with a tool provided by Wager et al. [27]. A bootstrapping procedure was applied to obtain an unbiased estimate of the indirect effect and a 99% confidence interval (CI). Specifically, the indirect effect was calculated by sampling with replacement where the sample size was equaled to the original one. This procedure was repeated 5000 times, which resulted in a distribution of the indirect effect. If 99% CI does not contain zero, the indirect effect is considered significant (i.e., p < 0.01). In the analysis, age and gender were treated as covariates.

### 2.5 Effects of head motion on neural correlates of impulsivity

#### 2.5.1 Data preprocessing

Anatomical and resting-state functional data were preprocessed using both FSL (http://www.fmrib.ox.ac.uk/fsl) and AFNI (http://afni.nimh.nih.gov). The first 4 volumes were discarded for MRI signals to reach a steady state. Preprocessing steps of fMRI data included spatial Gaussian smoothing (FWHM  =  6 mm), motion correction, intensity normalization, and removing linear trends. In addition, nuisance signals (i.e., six motion parameters, signals derived from cerebrospinal fluid and white matter masks after segmentation as well as global signals) were removed from the time courses using the general linear model.

#### 2.5.2 Amplitudes of low frequencies fluctuation (ALFF)

Recently, resting-state fMRI has emerged as a powerful approach for discovering neural signatures of both neurological and mental disorders (e.g., [28]). In the present study, we focused on the low frequency fluctuations (LFFs, 0.01-0.10 Hz) in the blood oxygen level-dependent (BOLD) signal at rest. Specifically, we employed an increasingly popular measure of LFFs, which is referred to as ALFF. ALFF reflects the strength of low frequency fluctuations of the brain under resting state. Among a variety of neural metrics in resting-state fMRI, ALFF becomes increasingly popular in identifying neural correlates of disorders such as ADHD [29] and autism [30]. In this study, ALFF values were calculated for each voxel based on a standard procedure [29]. Specifically, the time courses were first converted to the frequency domain using the fast Fourier transform. Then, the square root of power spectrum at each frequency was averaged across a frequency band of 0.01–0.10 Hz. Because low frequency fluctuations are most prominent in the gray matter, following analyses were conducted on the gray matter mask that included the voxels with a probability higher than 0.25 in the Harvard-Oxford brain template of the gray matter mask. It is worth noting that the ALFF was calculated with fMRI data that had been preprocessed with standard procedure, including head motion correction.

#### 2.5.3 Statistical analysis

To examine possible effects of head motion on the neural correlates of impulsivity, we explored the association between the behavioral measure of impulsivity (i.e., Hyper/impulsivity scores) and the ALFF of the brain across ADHD children either with or without including head motion as a confounding factor in the general linear model. The statistical maps were corrected for multiple comparisons with clusters determined by Z > 2.3 voxel-wise thresholding and a family-wise error-corrected (FWE-corrected) cluster significance threshold of p < 0.05 [31].

## Results

### 3.1 Head Motion and Impulsivity in Healthy Adults

Study 1 examined the potential link between in-scan head motion and impulsivity. Impulsivity was indexed for each participant by the total BIS score, with higher values indicating a greater level of impulsivity. In-scanner head motion was estimated as the average motion of each brain volume relative to the preceding one as previous studies [8], [9] during DTI scan. The mean, SD, and reliability of each measure is mentioned in Table 1. Note that the reliability of in-scanner head motion was relatively high (i.e., 0.83), suggesting that it is unlikely random noise but may reflect a trait-like property of the participants (e.g., [9], [32]). To test this instinct, next we examined the correlation between participants' in-scanner head motion and their scores in impulsivity.

**Table 1:** Mean Scores (± SD) and Reliability Estimates for Each Task in the Study.

	Mean ± SD	Reliability
In-scanner Head Motion (mm, n = 566; from DTI data in adults)	0.28±0.05	0.83
BIS Impulsivity (n = 566)	62.16±8.45	0.73
Attention	10.36±2.03	0.38
Instability	6.75±1.57	0.51
Motor	12.90±3.01	0.66
Perseverance	7.56±1.55	0.24
Self-control	12.33±2.63	0.57
Complexity	12.27±2.19	0.38

	ADHD (n = 93)	TDC (n = 124)
In-scanner Head Motion (mm; from resting-state fMRI data in children)	0.11±0.04	0.09±0.03
Hyper/impulsivity	22.38±6.45	13.54±3.61

The reliability of In-scanner head motion was Spearman-Brown-corrected split-half reliability, and that of BIS Impulsivity was Cronbach's alpha for items in the questionnaire. Note that the reliabilities of the components of impulsivity (e.g., self-control) were medium, possibly because the number of items for each component was small (e.g., 6 items for self-control). ADHD: attention deficit hyperactivity disorder; TDC: typically-developing controls; BIS: Barratt impulsiveness scale.

**Figure 1:**
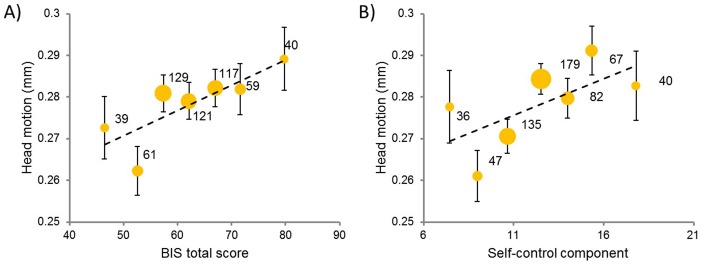
Binned scatter plots between in-scanner head motion and impulsivity indexed by (A) BIS total score, and (B) self-control component score. To avoid overlap for participants with similar scores, participants are binned into groups on the basis of impulsivity scores. The size of dots indicates the number of participants in the groups. Error bars indicate standard error of the mean (s.e.m).

We obtained a positive correlation between the magnitude of in-scanner head motion and self-reported impulsivity scores across participants (r  =  0.10, p  =  0.02; Spearman rho  =  0.10, p  =  0.02) (Fig. 1a). Further correlation analyses showed that among the 6 components of BIS impulsivity, only the self-control component contributed significantly to the association (r  =  0.14, p  =  0.001, Bonferroni corrected; Spearman rho  =  0.15, p < 0.0005; Fig. 1b), whereas the rest of the components were not correlated with in-scanner head motion (cognitive complexity: r  =  0.02, p  =  0.56; attention: r  =  0.08, p  =  0.07; cognitive instability: r  =  0.07, p  =  0.10; motor: r  =  0.07, p  =  0.12; perseverance: r  =  −0.001, p  =  0.99). Therefore, participants who tended to act without an appropriate amount of deliberation were more likely to have a larger amount of head motion during scanning, and such association was mainly due to difficulty with self-control. To test whether the motion-impulsivity correlation reported here was reliable, we randomly split the dataset into two halves (N  =  283 for each sub-dataset). We observed the motion-impulsivity correlation in both sub-datasets (Sub-dataset 1: r  =  0.13, p  =  0.027; Spearman rho  =  0.17, p  =  0.005; Sub-dataset 2: r  =  0.14, p  =  0.018; Spearman rho  =  0.13, p  =  0.030), suggesting that the association is reliable.

### 3.2 Impulsivity as a Mediator of ADHD-TDC Group Difference in Head Motion

The dataset for Study 2 comes from the ADHD-200 Consortium, which consisted of resting-state fMRI data and clinical behavioral data collected from ADHD and TDC. Impulsivity was assessed by the hyperactivity/impulsivity subscale of the ADHD-RS IV, and their in-scanner head motion was estimated from the resting-state fMRI data with the same procedure used in Studies 1.

As expected, children with ADHD scored significantly higher in impulsivity than those in the TDC group (t [215]  =  12.9, p < 0.001, two-tailed), and in-scanner head motion was significantly larger in children with ADHD than in children in the TDC group (t [215]  =  4.03, p < 0.001, two-tailed) (Table 1). In addition, the correlation between head motion and impulsivity was observed in the children dataset (r  =  0.34, p < 0.001). Similar patterns were observed in both ADHD children (r  =  0.26, p  =  0.01; Spearman rho  =  0.22, p  =  0.03) and TDC children (r  =  0.15, p  =  0.10; Spearman rho  =  0.19, p  =  0.03).

**Figure 2:**
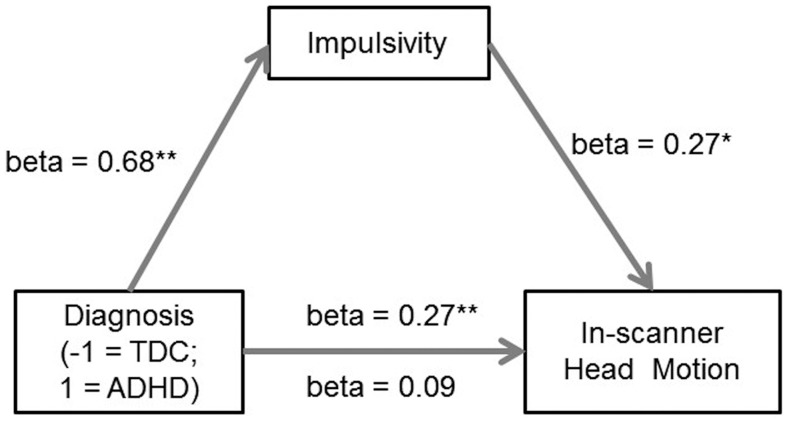
Impulsivity as a sufficient mediator of the difference in head motion between ADHD and TDC in the mediation analysis. Path coefficients are shown next to arrows indicating each link in the analysis. For the group difference in head motion, the value above the arrow indicates the zero-order correlation, and the value below the arrow represents the correlation after controlling the mediator of impulsivity. All values represent standardized betas. * indicates p < 0.01, ** indicates p < 0.001, two-tailed. ADHD: attention deficit hyperactivity disorder; TDC: typically developing children.

Furthermore, the mediation analysis [26] indicated that the difference between groups in head motion could be largely accounted for by the difference in impulsivity. In this model, the participant group (ADHD versus TDC) was the predictor, impulsivity was the mediator, and in-scanner head motion was the outcome. The mediation analysis showed that the difference in head motion between ADHD and TDC became insignificant (beta  =  0.09, p  =  0.35) after their association (beta  =  0.27, p < 0.001) was adjusted by the mediator of impulsivity (Fig. 2). Bootstrap simulation (n  =  5000) further confirmed that the indirect effect through impulsivity was significant (p < 0.01) with a 99% confidence interval of 0.0189 to 0.42. In other words, the difference between groups in the psychological trait of impulsivity accounted for the difference in head motion between children with ADHD and children in the TDC group.

### 3.3 Effects of Head Motion on Neural Correlates of Impulsivity

To deal with differences in head motion between participant groups, several groups have proposed to include head motion as a confounding variable in across-subject analyses [8], [21]. However, given the above-mentioned link between head motion and impulsivity, the regression approach may reduce the power to detect neural correlates of impulsivity. To demonstrate this possibility, in the third study we examined how head motion affected the identification of impulsivity-related cortical regions in the ADHD children. To do this, we correlated the behavioral measure of impulsivity and the ALFF of the brain, the strength of low frequency neural fluctuations under a resting state [29]. Head motion was either included as a confounding factor in the general linear model or not for the across-subject analysis.

**Figure 3:**
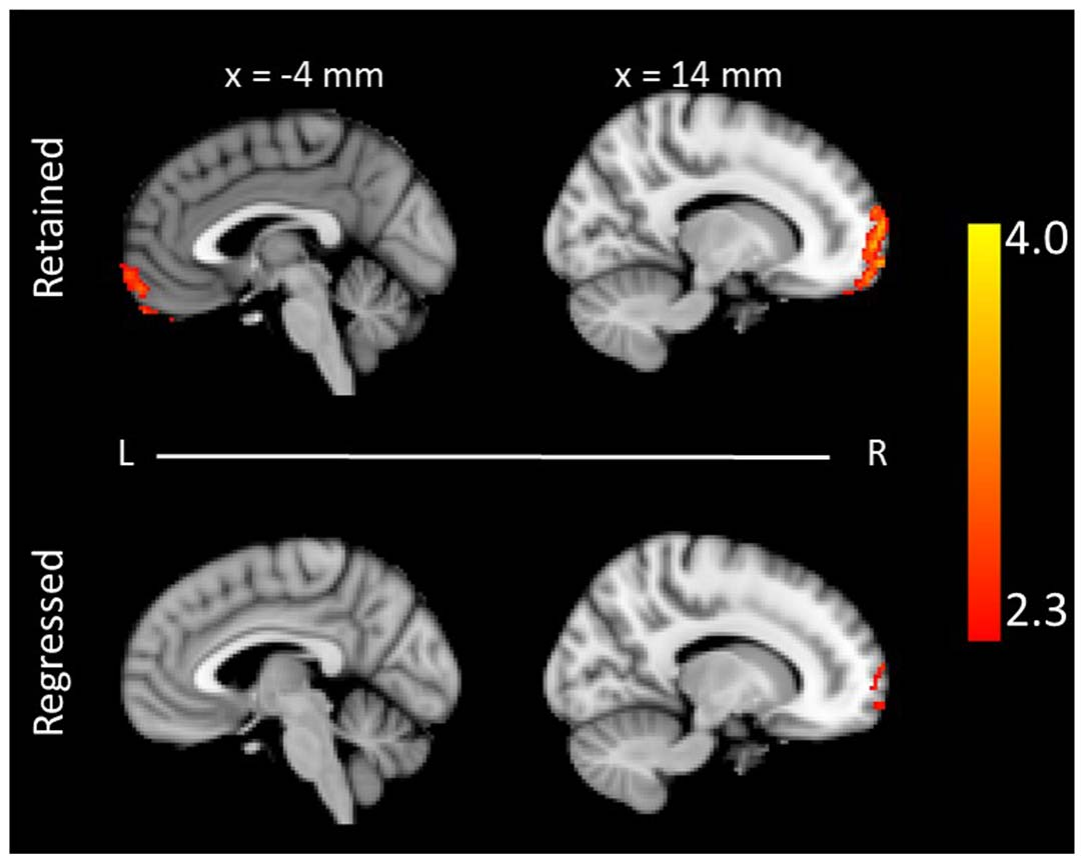
The neural correlates of impulsivity in the ADHD children. The ALFF value of the bilateral orbital frontal cortex and prefrontal cortex was positively correlated with the behavioral measure of impulsivity when the variable of head motion was either retained (top, Z > 2.3, p < 0.05, FWE corrected) or regressed out (bottom, Z > 2.3, uncorrected). The coordinate is in the MNI stereotactic space. ALFF: the amplitude of low-frequency fluctuation.

When the variable of head motion was not regressed out, we found a significant association between the ALFF of the brain and impulsivity in the right orbital frontal cortex (MNI coordinates: 12, 62, −16; Z-value  =  3.53, p < 0.05, FWE corrected) (Fig. 3, Top), which is a classic brain region related to impulsivity [33], [34], [35], [36], [37]. However, when head motion was considered as a confounding factor and then regressed out, the association was greatly weakened; indeed, no regions survived the multiple comparison correction (Fig. 3, Bottom).

## Discussion

Many researchers have attempted using MRI to identify neural signatures for disorders in the brain to improve the ability to diagnose such disorders, but inevitable head motion during MRI scanning poses difficulties in both data acquisition and interpretation. In this study, we elucidated the nature of head motion by correlating head motion with psychological traits in large samples. In two studies, we found that the variance of head motion was partly accounted for by the individual differences in impulsivity. Importantly, the association was observed across participant groups (children and adults) and across imaging modalities (resting-state fMRI and DTI), suggesting that the association between head motion and impulsivity can be observed across data modalities and ages. In addition, the final study suggests that the regression approach, which aims to deal with the issue on head motion in across-subject analyses, likely underestimated the effect of interest.

A recent study [19] has found that distant connectivity primarily in the default mode network significantly correlates with individuals' head motion during brain imaging, suggesting that head motion is an indicator of a specific cognitive control capacity in the individual brain. Our study extended this finding by showing a reliable association between impulsivity and in-scanner head motion. Because the association was apparently more prominent in children with ADHD, the effect of head motion might be particularly important in patient-control studies. Indeed, we demonstrated that the group difference in head motion between ADHD and TDC children was fully mediated by their difference in impulsivity. Together with recent studies [17], [19], our findings provide empirical evidence that the in-scanner head motion reflects trait-like properties of human participants, such as impulsivity, rather than just technical artifacts.

Note that the contribution of impulsivity to in-scanner head motion was small, possibly because of following reasons. First of all, in-scanner head does contain noise, which is evidenced by imperfect test-retest reliability of around 0.60 [9], [19], [38]. Therefore, together with the imperfect reliability of the measure on the impulsivity, the correlation coefficient of the motion-impulsivity is hardly larger than 0.60. Second, in this study the in-scanner head motion was measured at rest, and previous studies have shown that in the situation of low cognitive demands, the association between head motion and cognitive measures becomes weak [17]. Finally, the psychological trait of impulsivity is one of many factors that contribute to in-scanner head motion. Indeed, other psychological traits (e.g., conscientiousness, agreeableness, and anxiety), cognitive processes (e.g., high cognitive demands), and neurological/mental disorders (e.g., multiple sclerosis, schizophrenia) may also affect in-scanner head motion [17]. Having said this, the motion-impulsivity association cannot be simply ignored because of its small effect size. Instead, the association shall be taken into account especially in identifying neural signatures of some neurological/cognitive disorders because (1) the association is reliable across data modalities, subject populations, and ages, and (2) the association is likely more evident in population with impulsivity-related disorders (i.e., ADHD).

Recently, the neuroimaging field has become increasingly concerned about the confounding effect of in-scanner head motion on the interpretation of group differences in MRI measures, especially when the groups show differences in head motion. Accordingly, several groups have proposed to include head motion as a confounding variable in across-subject analyses to match head motion between groups [8], [21]. However, the regression approach may reduce the ability to detect the effect of interest [8]. For instance, previous studies have shown that the correlation coefficient between age and the functional connectivity between the MPFC and PCC reduces almost by 50% when head motion is regressed [8], [39]. One interpretation is that younger children move their head more intensively, which affects the quality of MRI data. Alternatively, head motion is associated with psychological or clinical traits (e.g., impulse control), cognitive processes [17], and even neurobiological substrates (e.g., functional connectivity [19]). Thus, regressing out head motion in the across-subject analysis likely underestimates the effect of interest. Consistent with the latter instinct, we found that the neural correlates of impulsivity indexed by ALFF greatly weakened when the regression approach was adopted. This result is in line with a recent finding that discarding data with severe motion artifacts, which is another approach proposed to deal with head motion, may introduce sampling bias [17].

On the other hand, it is unwise to conduct across-subject analyses without taking head motion into account, because head motion does interfere with the MR signals [1], [2], [3], and not modeling or removing head motion from MRI data surely produces spurious results [7], [8]. However, to differentiate the variance of interest (i.e., motion-associated endophenotypes) from the variance of error (i.e., motion-induced artifacts) is difficult with current techniques. Therefore, we suggest several ways of dealing with the head motion issue. First, results shall be presented both with and without head motion being regressed out in group-level analyses. If any significant difference is observed, the authors shall discuss such difference not only in terms of random noises but also in terms of meaningful variables such as psychological traits or the severity of disorders. Specifically, cortical regions that are modulated by head motion may likely provide clues on the type of cognitive or psychological variables underlying such modulation. Besides, replication in an independent sample is helpful, because motion-induced artifacts are usually random and not specific to brain regions. Third, an ideal solution is to minimize head motion artifacts during data acquisition so that there is no need to model head motion in data analysis. Several approaches have been proposed, one of which is to develop algorithms prospectively co-registering all images, as articulated previously [9], [17], [40]. Because this approach can prevent motion-induced artifacts on MR signals at the time of acquisition, it is likely superior to traditional realignment-based corrections. Finally, with advances in MRI acquisition techniques such as multi-band sequences [41], motion-induced artifacts can be effectively limited by increasing sampling rate [38], for example.

In sum, our study found that the variable of head motion is not simply a random factor, but reflects valuable information regarding individuals' psychological traits. Specifically, we found that a portion of variance in head motion could be explained by impulsivity, especially in the population with higher level of impulsivity. Thus, our study invites a broad investigation of whether other psychological traits (e.g., conscientiousness and agreeableness) or psychiatric symptoms (e.g., anxiety and claustrophobia) might contribute to in-scanner head motion. Such a finding may ultimately elucidate the relationship among in-scanner behaviors, psychological traits, and neural signatures of neurological disorders.
